# Comparative palatability and efficacy of two praziquantel isomers in rainbow trout infected by eyefluke

**DOI:** 10.1007/s00436-026-08733-y

**Published:** 2026-07-27

**Authors:** Shilu Huang, Kaan Kumas, Kurt Buchmann

**Affiliations:** Section for Parasitology and Aquatic Pathobiology, Department of Veterinary and Animal Sciences, Faculty of Health and Medical Sciences, University of Copenhagen, Stigbøjlen 7, Frederiksberg C, 1870 Denmark

**Keywords:** Cataract, Fish, Anthelmintics, Parasite

## Abstract

Praziquantel is a widely used anthelmintic in both human and veterinary medicine, but low palatability of the commonly used compound, which is a mixture (M) of two isomers (D and L forms), may result in rejection of oral treatments. Purification of the D-isomer of praziquantel allows a comparative evaluation of both palatability and efficacy of the isomers. We showed that rainbow trout (*Oncorhynchus mykiss*) preferred feed containing D-PZQ feed (60% acceptance) compared to M-PZQ feed (20% acceptance) (inclusion of 400 g PZQ/kg feed). Subsequent efficacy studies using eyefluke *Diplostomum pseudospathaceum* cercariae (*in vitro*) and experimentally infected rainbow trout carrying metacercariae (*in vivo*) demonstrated a strong antiparasitic effect of both PZQ-forms. *In vitro* assays documented that both M-PZQ and D-PZQ induced rapid and concentration-dependent mortality in cercaria (100% within 4 h in 0.125 mg PZQ/L), but with a significantly higher killing rate of of D-PZQ. *In viv*o assays (17 days feeding medicated feed) showed significant efficacy of both D-PZQ and M-PZQ. A slightly (non-significant) faster effect of D-PZQ may partly be ascribed to a combination of higher consumption of this isomer (due to its higher palatability) and higher efficacy. Cataract formation occurred in control fish but was prevented by both drug types indicating that metabolic and/or mechanical activities of live parasites are associated with cataract induction. This rainbow trout/eyefluke infection may serve as a model for non-piscine host-parasite systems as well.

## Introduction

Praziquantel (PZQ) is a broad-spectrum anthelmintic used to control trematode and cestode infections, both human and veterinary medicine (Andrews et al. 1983; Harnett 1988). It was also applied to treat fish infected with monogeneans (Schmahl et al. 1985; Buchmann et al. 1990; Partridge et al. 2014; Tang et al. 2022; Wan Sajiri et al. 2025), cestodes (Pool 1985) and trematodes (Bylund and Sumari 1981; Moser et al. 1986; Norbury et al. 2022). A major problem in freshwater and brackish water is diplostomosis (infections of fish with eyeflukes of the genus *Diplostomum*) (Buchmann 1986; Whyte et al. 1991; Buchmann and Bresciani 1997; Duan et al. 2021). The lifecycle includes a fish-eating bird as definitive host, a snail (family Lymnaeidae) as first intermediate host (with a sporocyst releasing cercariae) and fish as the second intermediate host carrying metacercariae in the lens. Following cercarial penetration of fish skin the parasite migrates as a tail-less diplostomulum to the eye lens and attains the metacercarial stage (Haarder et al. 2013; Locke et al. 2015). Their presence disrupts lens transparency (Shariff et al. 1980), leading to cataract formation, impaired vision (Seppälä et al. 2004), increased predation risk (Seppälä et al. 2006), reduced growth (Buchmann and Uldal 1994) and survival (Karvonen et al. 2004; Larsen et al. 2005).

Commercial PZQ-formulations consist of different isomeric forms, which can differ in physicochemical properties such as solubility, taste, and bioavailability (Meyer et al. 2009; Zhang et al. 2023). These properties may influence drug uptake, particularly when administered orally through medicated feed (Meyer et al. 2009; Shirakashi et al. 2012; Partridge et al. 2014, 2017, 2019). The relatively low palatability of PZQ is particularly important when drug mass distribution is needed in human populations exposed to parasitic diseases such as schistosomiasis (Boniatti et al. 2021; Mahende et al. 2021; Bedogni et al. 2023; N’Goran et al. 2023). However, low palatability raises special concerns not only in situations where oral treatment of humans infected with *Schistosoma* spp. is urgently needed (Meyer et al. 2009), but also when veterinarians treat dogs against cestodes (Courbet al. 2008; Schmid et al. 2010; Ciccone et al. 2024), and when infections in fish are treated orally. Thus, monogenean infections can be treated by PZQ in gilthead sea bream (Sitja-Bobadilla et al. 2006; Merella et al. 2024), in kingfish (Williams et al. 2007; Forwood et al. 2016) and silver perch (Forwood et al. 2013). Marine blood fluke infections in tuna can be treated with PZQ (Shirakashi et al. 2012) and freshwater blood flukes in carp (Hardy-Smith et al. 2012). Unless legally accepted for the specific purpose and host species the veterinarian must obtain dispensation of PZQ usage but documented efficacy studies may ease the process. Previous studies have focused mainly on the overall efficacy of PZQ (mixed D- and L-forms), while limited attention has been given to comparative evaluations of the specific PZQ isomers. Understanding whether different isomers exhibit distinct antiparasitic effects or differ primarily due to various degrees of food acceptance (or rejection) is essential for optimizing treatment strategies. The first part of the present study investigated the palatability of two drug forms when rainbow trout was offered feed containing them at different concentrations. The second part of this study aimed to compare the *in vitro* antiparasitic activity (against *D. pseudospathaceum* eyefluke cercariae) of PZQ (M-PZQ), which includes D and L isomers, and D-PZQ (pure D-isomer). The third part was designed as feeding experiment to assess *in vivo* efficacy against *D. pseudospathaceum* metacercariae infections in rainbow trout. Finally, we elucidated the potential mode of action by comparing metacercariae from treated versus non-treated fish, using parasitological methods. The results may have implications for human and veterinary medicine in general but are also directly applicable for farmed fish.

## Materials and methods

### Ethic statement

All experiments were conducted under license no. 2024-15-0201–01697 approved by the Experimental Animal Inspectorate, Committee for Experimental Animals, Ministry of Environment and Food, Denmark, and adhered to the ethical guidelines of the University of Copenhagen.

### Experimental fish

Rainbow trout were produced (hatched and reared) in a parasite free intensive trout-rearing facility based on recirculated municipal water at the Bornholm Salmon Hatchery (Nexø, Denmark) (Xueqin et al. 2012). They were then transported to our infection facility at the University of Copenhagen (Frederiksberg) (3 h transport time in oxygenated containers). Fish were kept in fish tanks with 120 L water volume, supplied with Frederiksberg commune municipal tapwater at 19℃. For palatability studies 15 larger trout (150–200 g total body weight) were used. For infection and subsequent feed-treatment trials we used 99 fish with TBW 3.5–4.5 g and total body length 6.5–7.6 cm.

### Praziquantel solutions

#### *In vitro* testing experiments

D-praziquantel (D-PZQ) (TL-230, CG552F-CH-180401) and M-praziquantel (Mixture PZQ containing both L- and D-form of Praziquantel) (TL-012, JYP-250306) was obtained from Tongli Biomedical Co. (Shanghai, China). Cercariae were exposed *in vitro* to a series of concentrations of two PZQ forms. Trials were conducted with 8 replicates for each group (octoplicates). Stock solutions of D-PZQ and M-PZQ were prepared by dissolving 2 mg D-PZQ (powder) in 1 mL of dechlorinated water containing DMSO (0.1%, v/v), resulting in a final concentration of 2000 mg/L. The solution was serially diluted with dechlorinated water in EP tubes to concentrations of 160, 80, 40, 20, 2, 1, 0.5, 0.25, 0.125, and 0.0625 mg PZQ/L. As controls were used solvent (0.1% DMSO as used in the stock solution) and pure water (negative control, 0 mg/L). Cercariae were exposed in a multiwell system (24 wells per plate, 500 µL solution per well) (Sarstedt, Nümbrecht, Germany). In each well a drug solution was added to an equal volume of water with cercariae, whereby parasites were exposed to half of the concentration added (80, 40, 20, 2, 1, 0.5, 0.25, 0.125, 0.0625, 0.031, 0.016, 0.007 and 0.003 mg PZQ/L with 0.05% (v/v) DMSO was used as solvent control.

### In feed for *in vivo* experiments

PZQ (D- and M-forms) feed was prepared separately. Feed particles (< 0.5 mm) were prepared by grinding (in mortar with pistil) pelleted fry feed (Inicio, Biomar, Denmark). The feed powder was then mixed with olive oil (6 µL per mg feed) and PZQ to concentrations of 10, 50, 100, 200 and 400 g PZQ/kg feed. Control feed was only mixed with olive oil. The oil allowed absorption of PZQ to feed particles. Prepared feed was formed into pellets (8 mm) and dried at low temperature (40 °C) overnight before it was presented to the fish for the palatability test. For the *in vivo* feed treatment trial targeting small fish, we used pelleted fry feed (0.5 mm), which was coated with olive oil (6 µL/mg feed) and either D-PZQ or M-PZQ. No grinding was performed.

### Palatability study

**Fig. 1:**
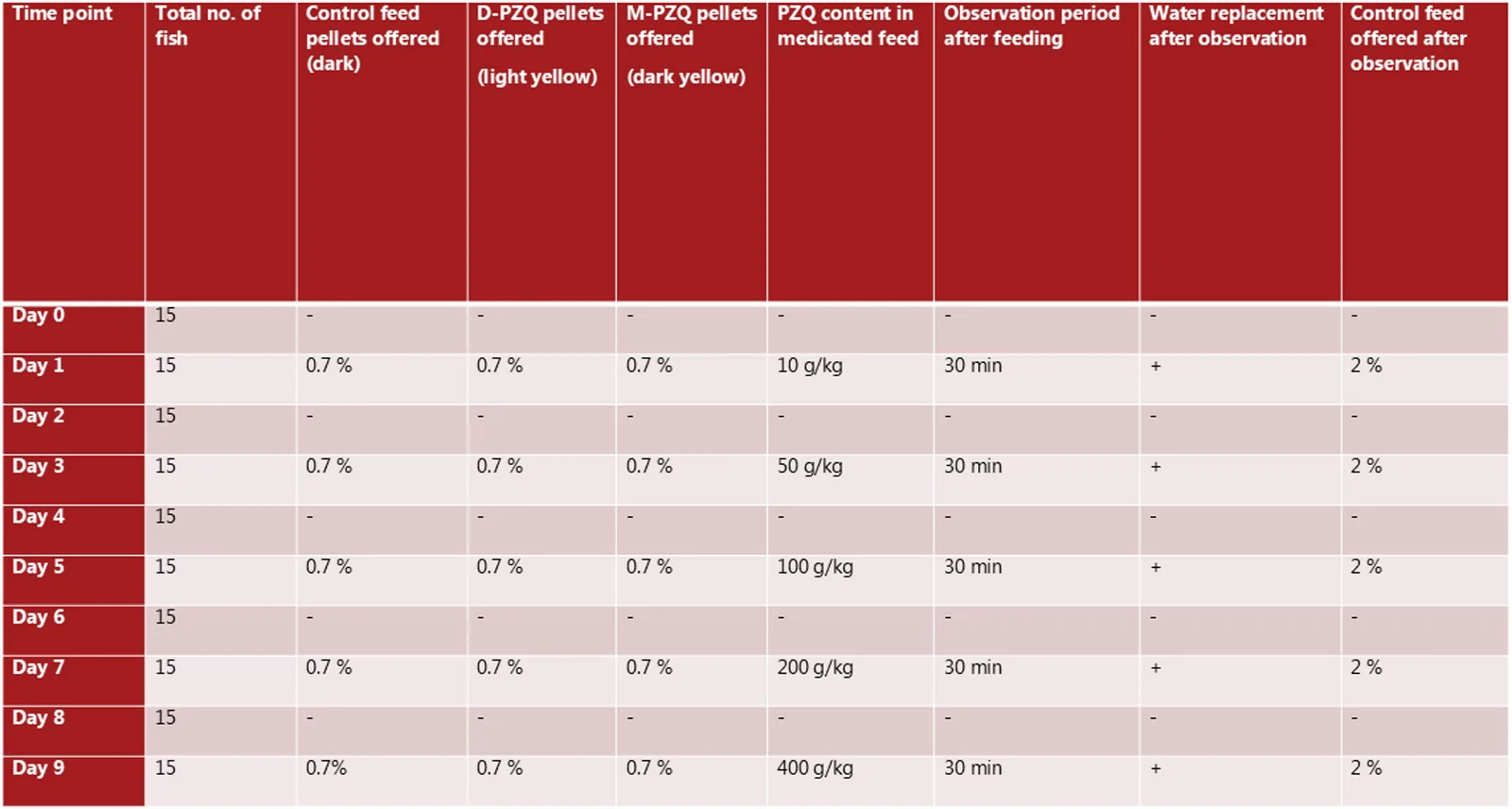
Experimental palatability set-up over 9 days. No. of replicates: 15. Selective feed intake was recorded after 30 min

The prepared feed was used to test rainbow trout feeding preferences regarding D-PZQ feed, M-PZQ feed and control feed without PZQ (olive oil only). Pathogen-free fish were allocated in individual tanks (water volume 60 L) for the feeding experiment (one fish in each tank, 15 replicates) (Fig. 1). Following a 24-hour period without feeding each fish was offered five pellets of each feed type (0.7% of total body weight TBW). They could be visually differentiated as M-PZQ (dark yellow), D-PZQ (light yellow) and control (dark brown). Fish were observed for 30 min, whereafter any feed rests (colour noted) were recorded and removed. The entire tank water volume was replaced, and the fish were then offered control feed (non-medicated) (2% TBW). The first day fish were offered the lowest PZQ concentration (10 g PZQ/kg feed), the third day 50 g PZQ/kg feed, the fifth day 100 g PZQ/kg feed, the seventh day 200 g PZQ/kg and the ninth day 400 g PZQ/kg feed. If feed pellets were left with a fish at the end of the 30 min observation period, it was considered rejected.

### Parasite source and identification

Infected pulmonated snails, *Lymnaea stagnalis*, were collected in the lake Søndersø (freshwater lake north of Copenhagen, Denmark) (55.7714478, 12.3652910) and transported to the laboratory. The infection status was confirmed after collection by placing the snail in a 100 mL beaker with filtered (0.45 μm) rainbow trout tank water (room temperature, daylight) to allow shedding (release of cercariae from infected snails). The snail was considered positive if *Diplostomum* cercariae were recorded under the stereomicroscope (7–40 magnifications) within 24 h. Parasites were then preserved in 96% ethanol for molecular identification, including DNA purification, PCR and sequencing of rDNA, the internal transcribed spacer (ITS) (Haarder et al. 2013; Duan et al. 2021) (see details below).

### Isolation of cercariae for experimental infection

The snail with confirmed infection was then maintained at room temperature in the beaker and fed fresh organic lettuce. Water was replenished daily with filtered fish tank water. Freshly released cercariae were collected by pipetting at regular intervals (5–8 h) and enumerated in Petri dishes under a dissection microscope. They were then used for *in vitro* exposure to PZQ and experimental infection of fish for *in vivo* PZQ treatment trials.

### Molecular identification of cercariae

Cercariae freshly released from the intermediate host snails were preserved in 96% ethanol at 4 °C. DNA extraction of the parasite carried out by using QIAamp DNA Mini Kit (Qiagen, Hvidovre, Denmark) according to the manufacturer’s instructions, with the exception that the final volume of AE buffer was 50 µL. PCR was performed using BioRad T100 Thermal Cycler (Bio-Rad Laboratories, Denmark) with 60 µL reaction volume containing 6 µL of 100 mM dNTP mix (Applied Biosystems™ GeneAmp™ dNTP Blend, Fisher Scientific, Slangerup, Denmark), 28.6 µL of DNase and RNase-free water (Fisher Scientific), 6 µL (each) of 10 mM forward and reverse primers (TAG Copenhagen, Denmark), 1.8 µL of 50 mM MgCl_2_, 0.6 µL of DNA Polymerase, 6 µL of 10X Reaction buffer (all three BIOTAQ DNA Polymerase, Saveen & Werner ApS, Jyllinge, Denmark), and 5 µL of parasite DNA. The ITS region encoding ribosomal RNA (suitable for eyefluke species differentiation) was targeted using the forward primer PDG_18S_F5 (5′-CGATAACGAACGAGACTC-3′) according to (Wan Sajiri et al. 2024) and reverse primer NC2 (5′-TTAGTTTCTTTTCCTCCGCT-3′) according to (Gasser et al. 1993). The PCR protocol comprised pre-denaturation at 94 °C for 5 min, followed by 40 cycles of denaturation at 95 °C for 30 s, annealing at 54 °C for 30 s and elongation at 72 °C for 2 min, followed by 72 °C for 7 min. The PCR product was then visualized by 2% agarose gel electrophoresis and purified by using Illustra™ GFX™PCR DNA and Gel Band Purification Kit according to the manufacturer’s instructions (VWR, Søborg, Denmark). Sequencing was performed at Macrogen Europe, the Netherlands, and data analysed using the software CLC-Main Workbench v20.0.0.4 (QIAGEN) and complete sequences submitted to GenBank.

### *In vitro* exposure of cercariae to PZQ

*D. pseudospathaceum* cercariae were transferred to a 24-well plate. The number varied from 15 to 20 cercariae/well with 0.5 mL water, whereafter PZQ solutions were added in equal volumes securing exposure of parasite larvae as defined above. Water with 0.05% DMSO and 0 mg/L served as negative controls. Eight replicates were set up for each concentration of M-PZQ and D-PZQ. The motility and viability of cercariae in the individual wells were monitored under a dissection microscope every 10 min for 4 h. The number of live cercariae (moving or showing motility when stimulated by a needle) at each time point was recorded.

### Experimental infection of fish

**Fig. 2:**
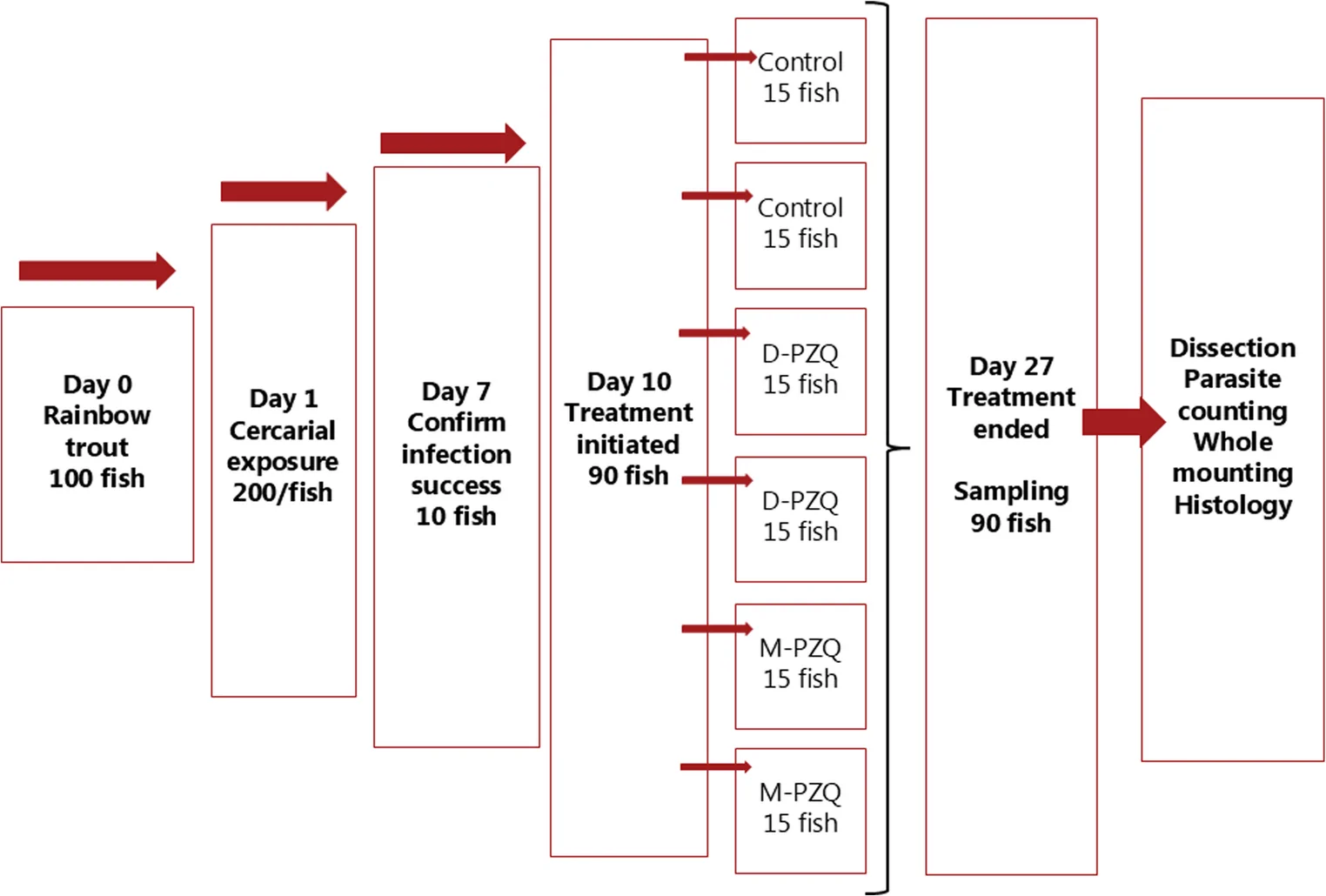
Experimental design of the *in vivo* PZQ feed trial. The study was duplicated and oral treatment performed over 17 d

A total of 100 rainbow trout were kept in 40 L aerated municipal water at 19 °C, whereafter freshly harvested cercariae (200/fish) were added to the tank (Fig. 2). Each fish was exposed to cercariae for 1 h. Then fish were moved to 120 L water without cercariae. Seven days after exposure 10 fish were examined to confirm the presence of *D. pseudospathaceum* in eye lenses (Buchmann 2007).

### PZQ treatment of eyefluke infected fish (*in vivo* study)

Based on the feed preference study (see below) 200 g PZQ/kg feed was selected as the experimental concentration to be used for *in vivo* oral treatment trials against diplostomosis because both feed types were eaten to some extent. The experiment was carried out in duplicate. Each experimental group (both for D-PZQ M-PZQ and control group) consisted of 30 rainbow trout, which were evenly distributed into two tanks (15 fish per tank) (Fig. 2). All fish were maintained in 80 L water at 19 °C in continuous light throughout the experiment. From 10 to 27 dpi fish were treated orally. Fish were fed (non-medicated control, D-PZQ, M-PZQ) based on a commercial diet (Inicio, Biomar, Denmark) at 2% of their body weight (TBW) daily, divided into two feeding time points (at 9:00 and 16:00 h). Thirty minutes after feeding, any remaining feed was removed, and 50% of the tank water was replaced with fresh tap water (Fig. 2).

### Parasitological examination of infected fish

On day 7 of the trial, 10 fish were randomly selected pre-treatment for evaluation of *Diplostomum* infection and body weight and length measurements. The lens and *corpus vitreum* of each sampled fish was examined for infection and activity (movement, contraction, extension) of metacercariae present was assessed. On day 17 following initiation of treatment (27 dpi) the same measurements and examinations were performed on parasites from all remaining fish.

### Examination of metacercarial morphology (whole mounts)

On day 17 of the feed-treatment trial (27 dpi), eyes from fish were dissected, lenses isolated, metacercariae enumerated, isolated, transferred to 10 mL 70% ethanol and stored at 4 °C for subsequent whole-mounting (*in toto* preparation) in glycerine gelatine and microscopic observations (Buchmann 2007). Morphological characteristics including oral sucker, ventral sucker, pharynx, intestine, posterior bladder and tegument integrity were used to describe parasite morphology and integrity.

### Examination of metacercarial morphology (histology)

To evaluate any drug effects on organ structure in parasites we performed a histological analysis. On the same day 17 of the feed-treatment trial, two eyes dissected from three fish from each group were fixed in neutral buffered formalin (NBF) for 24 h and prepared for histology. This included standard dehydration in graded ethanol series and paraffin embedding. Sections of 4 μm were cut using a Leica microtome, deparaffinized, rehydrated, routinely stained with H&E and mounted in Aquatec (Merck, Germany). Light microscopical analysis focused on organ structure and parasite parenchyma integrity.

### Statistics

All statistical comparisons and figures were conducted using GraphPad Prism version 11.0.0. (San Diego USA). To assess normality and log-normality, the Kolmogorov-Smirnov test was performed. In cases that followed a normal distribution, one-way ANOVA and two-way ANOVA with Šídák’s multiple comparisons tests were applied. If data did not follow a normal distribution, a nonparametric two-tailed Mann-Whitney test or Kruskal-Wallis test with multiple comparisons was used. A general probability level of 5% was established for all statistical tests but p-values obtained are shown for information.

## Results

### Parasite identification

The cercariae were identified as *Diplostomum pseudospathaceum* by molecular biology methods targeting rDNA (ITS) region. The PCR product was 1688 bp long, including primer binding sites (GenBank acc. No. PX675814). Blast analysis at NCBI confirmed identity as *D. pseudospathaceum*, with the closest identities from 99.92% to 99.76% (GenBank acc. Nos. OK329927.1 and MW000985.1, respectively).

### Trout preference for feed types

**Fig. 3:**
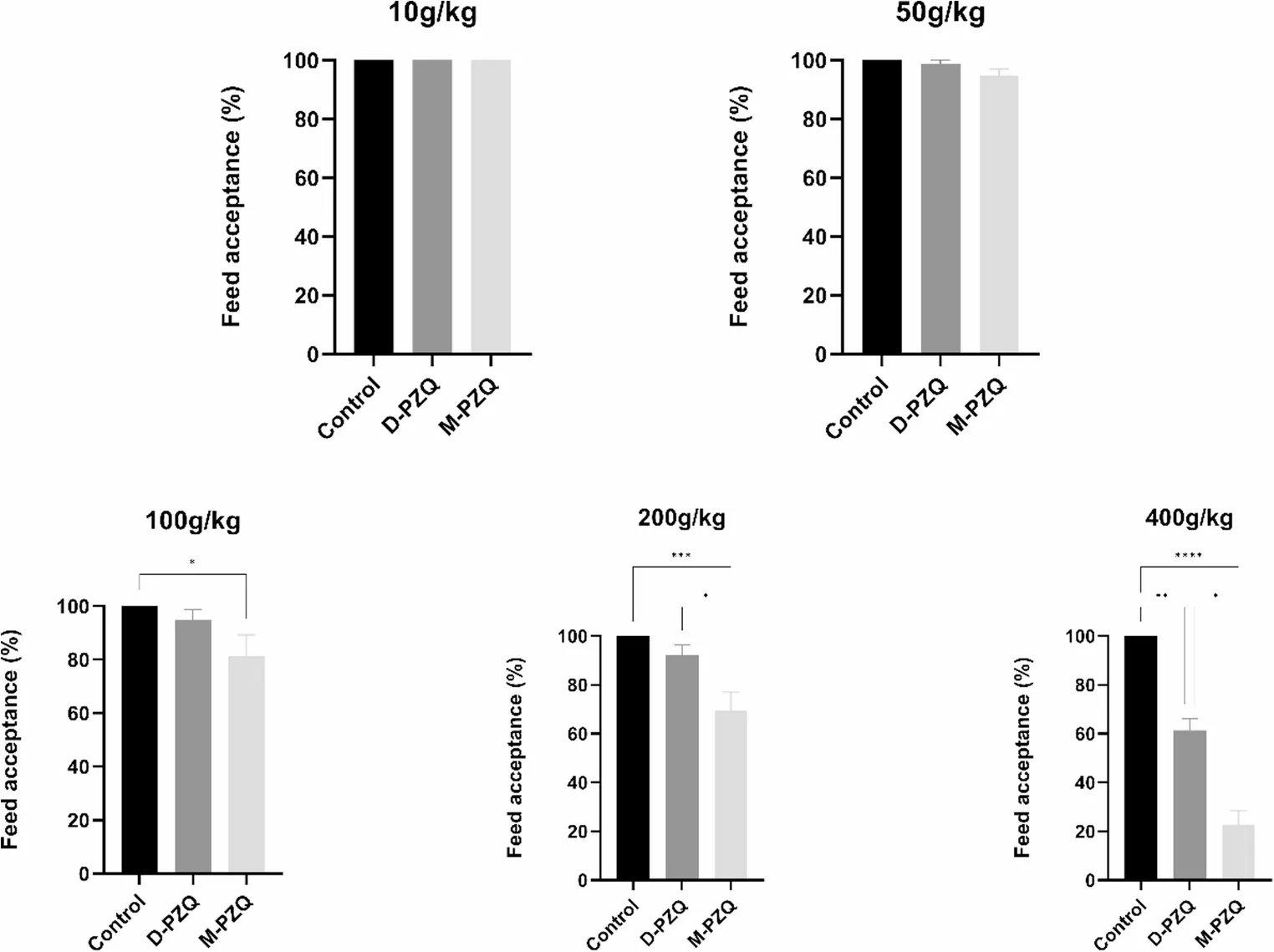
Palatability expressed as feed acceptance in percentage within 30 min. Rainbow trout were offered feed with different concentrations of D-PZQ and M-PZQ (control feed without any PZQ). Differences between treated groups were tested by Kruskal-Wallis with Dunn’s multiple comparisons test. *: *P* < 0.03, **: *P* < 0.01, ***: *P* < 0.0002, ****: *P* < 0.0001

Rainbow trout showed significantly different feed preference when offered a high concentration (from 100 to 400 g PZQ/kg) of the two PZQ-isomers in feed (Fig. 3). Intake of M-PZQ medicated feed with a content higher than 100 g M-PZQ/kg significantly decreased (*p*<0.01) when compared to intake of control feed and D-PZQ. The D-PZQ medicated feed intake was significantly reduced (*p*<0.01) but at a higher concentration (400 g D-PZQ/kg), when compared to the control feed.

### *In vitro* effects of PZQ on *D. pseudospathaceum* cercariae

**Fig. 4:**
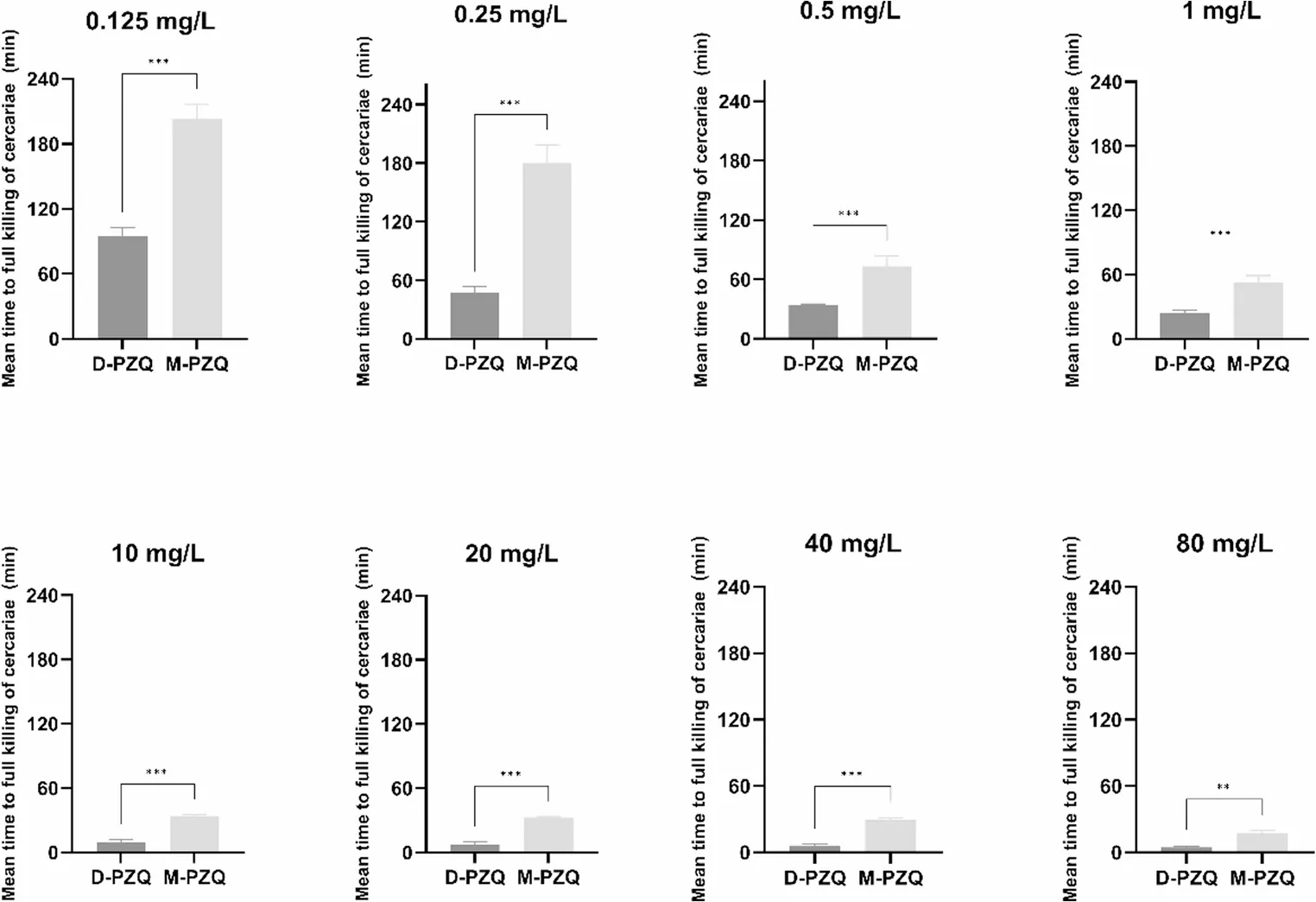
*In vitro* exposure of cercariae to D-PZQ and M-PZQ. Time (min) to complete killing of cercariae obtained within 4 h at different concentrations. Differences between PZQ types (for each concentration) were tested by two tailed Mann Whitney test. **: *P* < 0.002, ***: *P* < 0.0005

M-PZQ and D-PZQ showed a concentration-dependent effect on cercariae (Tables 1 and 2). Concentrations of both PZQ isomers above 0.625 mg/L killed all parasites within 4 h. However, the killing rate at concentrations from 0.0626 mg/L to 80 mg/L differed significantly, as D-PZQ killed cercariae significantly faster compared to M-PZQ (Fig. 4).

**Table 1:** *In vitro* assays. Mean mortality of *D. pseudospathaceum* cercariae exposed to different concentrations of D-PZQ for 4 h. Total numbers represent a total of 8 replicate assays. Mean mortality of eight replicates. Partial killing observed at 4 for the lowest concentrations (< 0.0625 mg/L). Significant difference between mortality in groups were calculated by Kruskal-Wallis with Dunn’s multiple comparisons test, and all groups were compared against 0 (Control). *: *P: 0.001*, **: *P: <0.0001*

Groups and concentrations (mg/L)	Total number of cercariae	Total number of live cercariae	Total number of dead cercaria	Mean mortality (SD) 4 h post-exposure
0 (Control)	144	144	0	0.0 ± 0.0
DMSO (0.05)	147	147	0	0.0 ± 0.0
0.003	127	119	8	6.9 ± 5.7
0.007	132	99	33	24.9 ± 12.4
0.016	133	79	54	40.0 ± 8.6
0.031	134	37	97	71.5 ± 17.5
0.0625	146	6	140	96.2 ± 5.4*
0.125	124	0	124	100.0 ± 0.0**
0.25	127	0	127	100.0 ± 0.0**
0.5	158	0	158	100.0 ± 0.0**
1	148	0	148	100.0 ± 0.0**
10	153	0	153	100.0 ± 0.0**
20	155	0	155	100.0 ± 0.0**
40	151	0	151	100.0 ± 0.0**
80	146	0	146	100.0 ± 0.0**

**Table 2:** *In vitro* assays. Mean mortality of *D. pseudospathaceum* cercariae exposed to different concentrations of M-PZQ for 4 h. Total numbers represent a total of 8 replicate assays. Mean mortality of eight replicates. Partial killing observed at 4 h for the lowest concentrations (< 0.0625 mg/L). Significant difference between mortality in groups were calculated by Kruskal-Wallis with Dunn’s multiple comparisons test, and all groups were compared against 0 (Control). *: *P: 0.001*, **: *P: <0.0001*

Groups and concentrations (mg/L)	Total number of cercariae	Total number of alive cercariae	Total number of dead cercaria	Mean mortality (SD) 4 h post-exposure
0 (Control)	160	160	0	0.0 ± 0.0
DMSO (0.05)	159	159	0	0.0 ± 0.0
0.003	146	140	6	4.0 ± 3.3
0.007	133	101	32	25.3 ± 9.1
0.016	126	51	75	58.9 ± 13.1
0.031	122	42	80	65.4 ± 12.8
0.0625	133	19	114	86.8 ± 10.6*
0.125	143	0	143	100.0 ± 0.0**
0.25	136	0	136	100.0 ± 0.0**
0.5	139	0	139	100.0 ± 0.0**
1	137	0	137	100.0 ± 0.0**
10	143	0	143	100.0 ± 0.0**
20	148	0	148	100.0 ± 0.0**
40	150	0	150	100.0 ± 0.0**
80	156	0	156	100.0 ± 0.0**

### Viability of metacercariae in lenses (*in vivo* study)

**Fig. 5:**
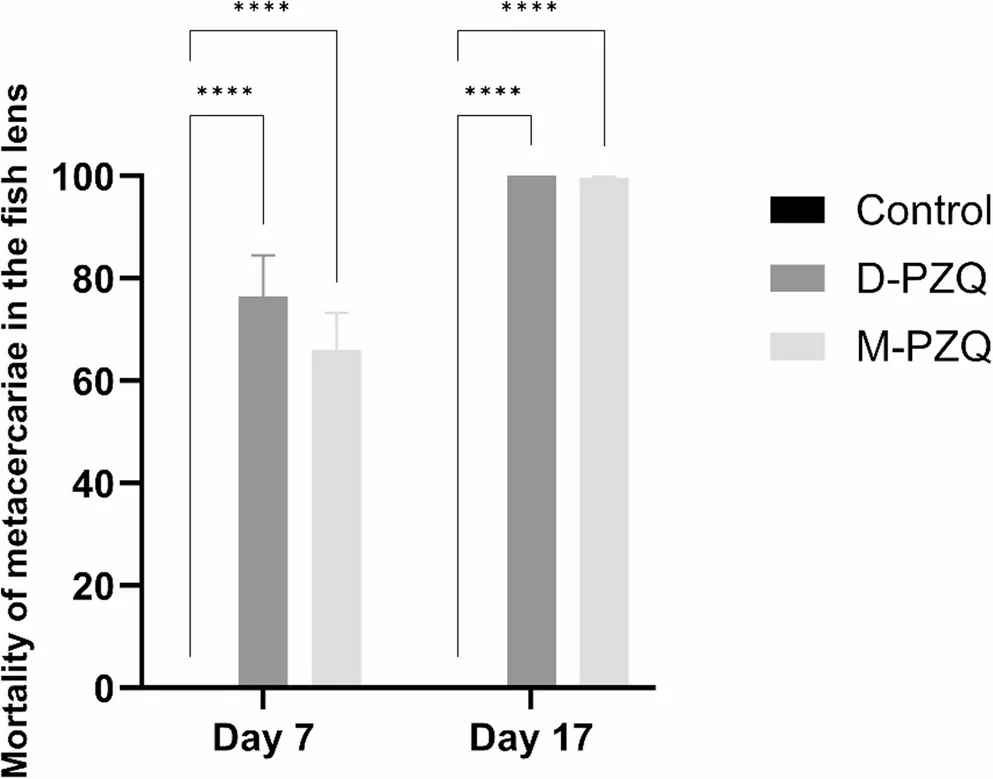
Mortality of metacercariae in fish lenses examined on 7th and 17th day post-treatment with D-PZQ and M-PZQ. Differences were tested by two-way ANOVA with Šídák’s multiple comparisons test, all groups tested each against each other at each time point. ****: *P* < 0.0001

Both medicated feed types had an impact on survival of metacercariae in lenses of rainbow trout, although D-PZQ again showed a non-significant trend for higher efficacy (Fig. 5). On day 7 the two medicated feed types had induced parasite mortalities of 75% (D-PZQ) and 65% (M-PZQ), respectively. By day 17 D-PZQ fed fish exhibited parasite mortality of 100% and M-PZQ 99%. Microscopic examination of infected fish lenses revealed cataracts (opaque lens matrix) in half of the fish in the control group already at day 7 during the feeding experiment. At day 17 (27 dpi) cataracts were observed in all lenses of fish in the control group. Both medicated feeds prevented cataract formation.

### Effects on parasite morphometrics (whole mounts and histology)

**Fig. 6:**
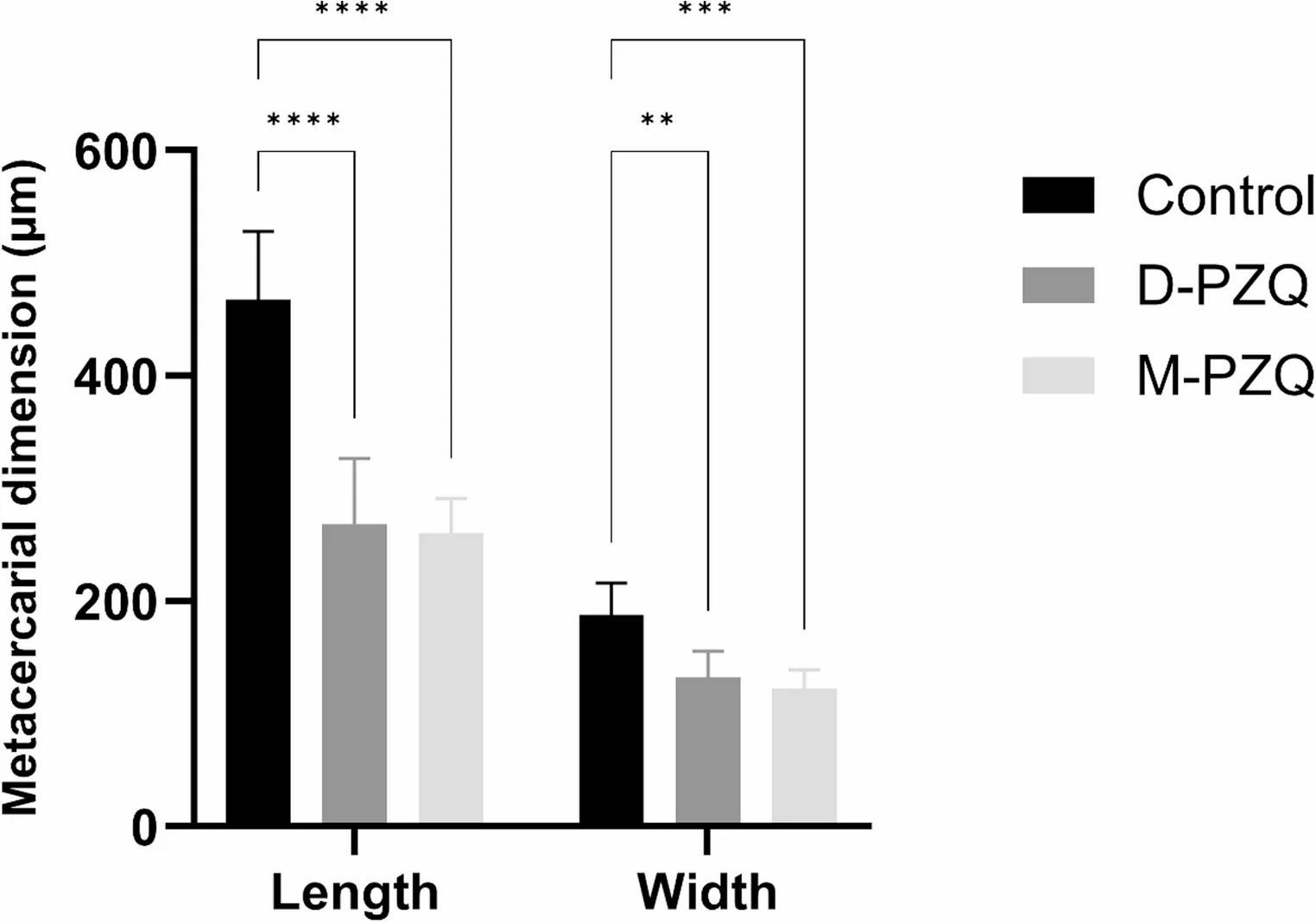
Dimensions (length and width in µm) of metacercariae isolated 17 d post-treatment from each treatment group (D-PZQ, M-PZQ, control). Based on glycerin-gelatine samples (whole mounts) examined with light microscope. Significant differences calculated with two-way ANOVA with Šídák’s multiple comparisons test. **: *P* < 0.003, ***: *P* < 0.0004, ****: *P*:<0.0001

Metacercariae collected on day 17 post-treatment were whole-mounted and measured (length and width) and when compared to parasites in the untreated group, both PZQ feed treatments significantly reduced metacercarial length and width (*p* < 0.01) (Fig. 6).

**Fig. 7:**
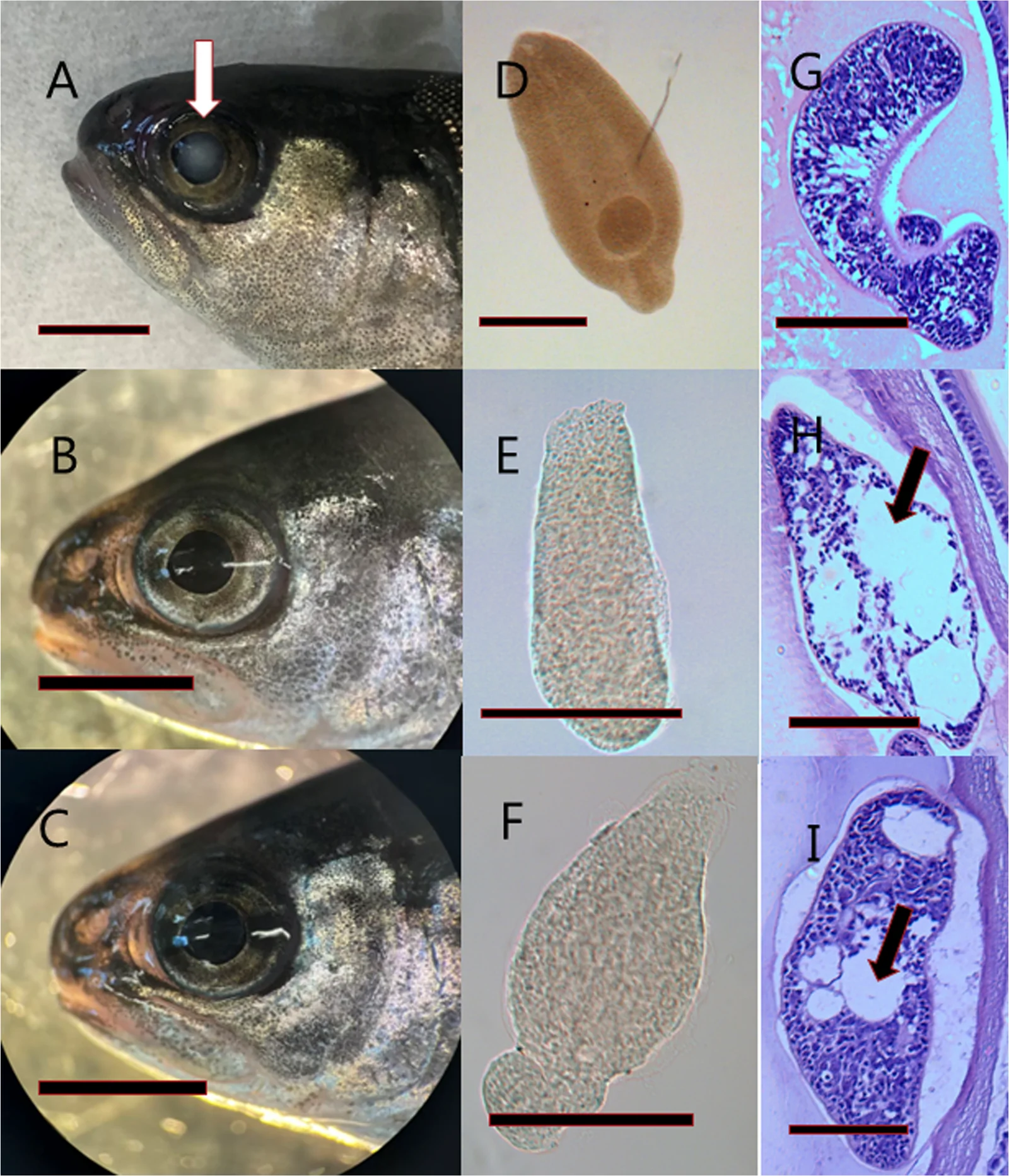
Effects associated with oral PZQ treatment of *D. pseudospathaceum* infected rainbow trout by D-PZQ and M-PZQ (in feed). **A**-**C**: Cataract (white arrow) formation in non-treated rainbow trout (**A**) compared to D-PZQ (**B**) and M-PZQ (**C**) treated fish. Scale bar: 5 mm. **D**-**F**: Integrity and shape of metacercariae: Control (**D**), D-PZQ treated €, M-PZQ treated (**F**) (whole mounts). Scale bar: 150 μm. **G**-**I**: Disrupted (black arrow) parasite parenchyma in treated metacercariae (histology). Scale bar: 50 μm

Thus, the PZQ treatment induced a clear disintegration and shrinking of metacercariae when compared to control parasites (Fig. 7), but no statistically significant difference in metacercarial size was observed between the two treatment groups (D-PZQ and M-PZQ). Regarding the integrity of eye flukes in fish lenses, light microscopic showed that metacercariae in the non-treated control group had intact organs, including distinct bladders, oral suckers, and ventral suckers. In contrast, metacercariae from lenses in fish fed both types of PZQ feed treatment groups exhibited disrupted organ structures and suckers, while intestine and bladder could not be recognized. Histological analysis showed compact parasite parenchyma and intact suckers in non-treated metacercariae, but both D-PZQ and M-PZQ treated parasites showed disrupted parenchyma with large areas of disintegrated tissue (Fig. 7).

**Fig. 8:**
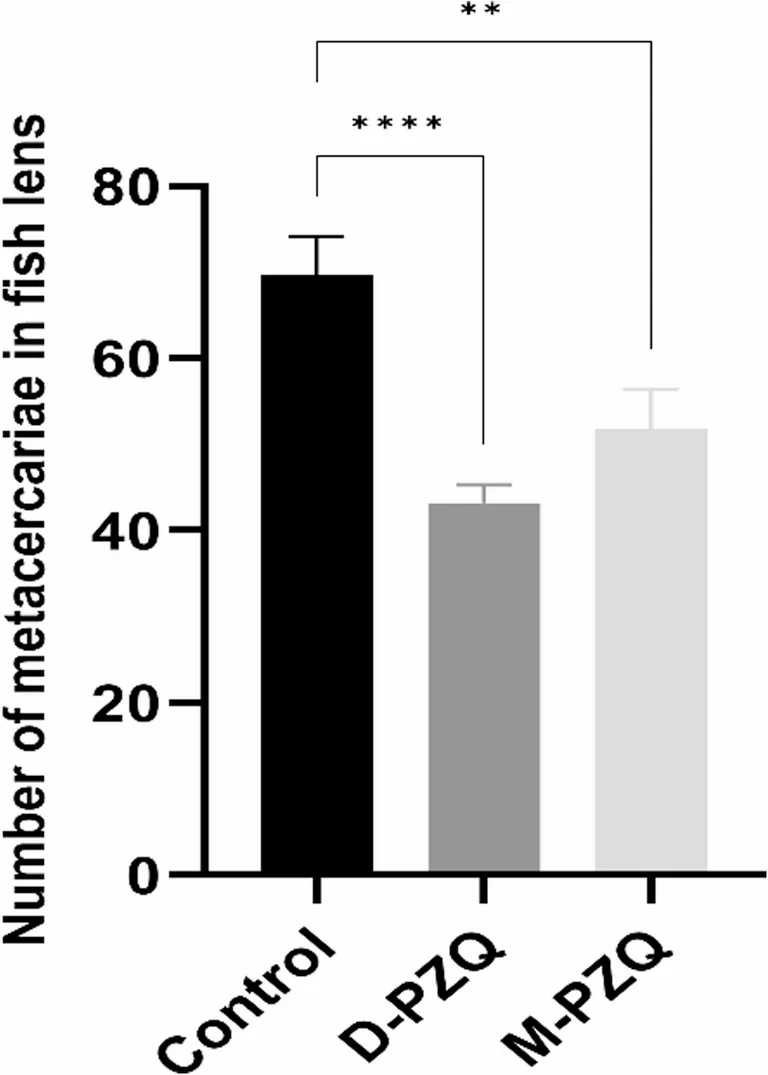
Total number of metacercariae counted in fish lenses on day 17 post- treatment. Significant differences calculated by one-way ANOVA with Šídák’s multiple comparisons test. **: *P* < 0.006, ****: *P*:<0.00001

When enumerating metacercariae in the three experimental groups 17 d after starting of the feeding experiment it was found that the infection intensity in controls was significantly higher when compared to the two treatment groups (D-PZQ and M-PZQ) (Fig. 8).

## Discussion

Oral treatments of fish evidently depend on successful ingestion of the medical compounds in-feed. Palatability is therefore a crucial factor when deciding a treatment regime e.g. in aquacultural enterprises. The present feeding preference experiments demonstrated that rainbow trout may reduce intake of M-PZQ medicated feed when offered at high concentrations. This compound, composed of a D- and a L-form has a bitter taste (Meyer et al. 2009), which may explain the partial rejection observed in the present study. In this study we investigated if the reduced palatability is due to the content of the L-form in the M-PZQ applied, as reduced consumption of medicated feed will limit overall drug intake and thereby diminish parasite elimination in infected hosts (Zhang et al. 2023). We showed that D-PZQ containing feed was better accepted by rainbow trout, which adds to the notion that D-PZQ has a better taste.

We suggest that the the improved acceptability of the D-PZQ may contribute to the slightly superior treatment results. Such an association has been noted in other host-parasite systems, such as in blood fluke treatments in Pacific bluefin tuna (Shirakashi et al. 2012). These findings indicate that efficacy differences observed during *in vivo* studies are not always attributable to intrinsic pharmacological differences between isomers, but in some cases due to mediated feed factors influencing intake and drug exposure.

This study confirms and extends previous findings demonstrating that praziquantel exhibits rapid and potent antiparasitic effects (Andrews et al. 1983; Buchmann et al. 1990; Charlotte and David 2018). Both M-PZQ and D-PZQ showed *in* *vitro* efficacy against *D. pseudospathaceum* cercariae, inducing mortality in a clear concentration-dependent manner, but we found a statistically significant difference between M-PZQ and D-PZQ under these *in vitro* conditions (cercarial exposure). Thus, D-PZQ showed a superior effect. At higher concentrations (20–80 mg/L), both M-PZQ and D-PZQ induced near-immediate paralysis and death of cercariae, whereas lower concentrations required longer exposure times to induce complete parasite mortality, confirming a concentration dependent action mechanism (Charlotte and David 2018; Greenberg 2005). This may involve disruption of calcium homeostasis, membrane integrity, and osmotic balance (Doenhoff et al. 2008; Shaw and Erasmus 1987). While our study did not specifically elucidate molecular pathways involved, our microscopic observations of whole mounts and histological evidence support a mechanism involving organ degradation, disruption of cellular integrity and organization.

*In vivo* treatment trials revealed significant differences in therapeutic outcomes for D-PZQ and M-PZQ. Both medicated feed types effectively reduced metacercarial survival and prevented cataract formation, confirming that praziquantel is able to reach the lens tissue following oral administration (Bylund and Sumari 1981). However, D-PZQ induced a non-significantly higher and faster parasite inactivation seen both after 7 and 17 days of feeding medicated feed. Light microscopic and histological analyses further supported the antiparasitic action of both isomers. Treated metacercariae displayed reduced size, disrupted organ and cellular architecture, indicating inhibited growth and degeneration as described in other parasite-host systems (Shaw and Erasmus 1987). Such structural damage likely compromises parasite viability and infectivity, contributing to reduced cataract formation and improved ocular health in treated fish.

The prevention of cataract formation in PZQ treated fish suggests that this pathological effect is caused by live metacercariae probably due to metabolic and/or mechanical activity. It is noteworthy that the number of metacercariae was significantly higher in non-treated control group. This suggests that some diplostomules were still migrating in trout towards the eye on day 10 post-infection, when feeding was initiated. These may have been killed during migration, whereby they would not have reached the eye at day 17 of PZQ treatment. Our results did detect differences in antiparasitic effects between D-PZQ and M-PZQ *in vitro*, but the difference seen during the *in vivo* trial may be due to a combination of feed palatability and drug efficacy. In all cases we emphasize the importance of feed palatability when designing oral antiparasitic treatments in aquaculture. Even compounds with equivalent *in vitro* efficacy may result in different therapeutic outcomes *in vivo* due to differences in voluntary drug intake, which again is caused by different palatability of the two isomers.

## Conclusion

This study demonstrated that rainbow trout prefer feed containing D-PZQ, when compared to feed with M-PZQ. However, both M-PZQ and D-PZQ possess strong antiparasitic activity against cercariae and metacercariae of *Diplostomum pseudospathaceum* and are thereby effective in controlling eye fluke infections in rainbow trout by both drug isomers, but the D-isomer showed a slightly faster killing of cercariae. While significant differences were observed between the two isomers *in vitro*, feeding with D-PZQ led to a slightly superior *in vivo* efficacy, achieving earlier and faster killing of all metacercariae. Both isomers prevented cataract formation. The observed difference in therapeutic performance is most likely attributable to a combination of different palatability of the two isomers and difference in effect. These findings highlight the critical role of host feeding behaviour in oral drug delivery and underscore the need to optimize medicated feed formulations for effective parasite control in aquaculture systems. Although performed in a fish host-parasite system, we suggest that these principles may be relevant for human and veterinary medicine as well.

## Data Availability

All data in this study are fully available on reasonable request
